# Defining the limits of plant chemical space: challenges and estimations

**DOI:** 10.1093/gigascience/giaf033

**Published:** 2025-04-04

**Authors:** Chloe Engler Hart, Yojana Gadiya, Tobias Kind, Christoph A Krettler, Matthew Gaetz, Biswapriya B Misra, David Healey, August Allen, Viswa Colluru, Daniel Domingo-Fernández

**Affiliations:** Enveda, Boulder, CO 80301, United States; Enveda, Boulder, CO 80301, United States; Enveda, Boulder, CO 80301, United States; Enveda, Boulder, CO 80301, United States; Enveda, Boulder, CO 80301, United States; Enveda, Boulder, CO 80301, United States; Enveda, Boulder, CO 80301, United States; Enveda, Boulder, CO 80301, United States; Enveda, Boulder, CO 80301, United States; Enveda, Boulder, CO 80301, United States

**Keywords:** cheminformatics, chemical space, natural products, plants

## Abstract

The plant kingdom, encompassing nearly 400,000 known species, produces an immense diversity of metabolites, including primary compounds essential for survival and secondary metabolites specialized for ecological interactions. These metabolites constitute a vast and complex phytochemical space with significant potential applications in medicine, agriculture, and biotechnology. However, much of this chemical diversity remains unexplored, as only a fraction of plant species has been studied comprehensively. In this work, we estimate the size of the plant chemical space by leveraging large-scale metabolomics and literature datasets. We begin by examining the known chemical space, which, while containing at most several hundred thousand unique compounds, remains sparsely covered. Using data from over 1,000 plant species, we apply various mass spectrometry–based approaches—a formula prediction model, a *de novo* prediction model, a combination of library search and *de novo* prediction, and MS2 clustering—to estimate the number of unique structures. Our methods suggest that the number of unique compounds in the metabolomics dataset alone may already surpass existing estimates of plant chemical diversity. Finally, we project these findings across the entire plant kingdom, estimating that the total plant chemical space likely spans millions, if not more, with most still unexplored.

## Background

Hundreds of thousands of species exist in the plant kingdom. Current estimates cover approximately 390,000 species, with a few thousand novel vascular plants being discovered every year [1]. Each plant produces thousands of primary and specialized metabolites for survival and environmental interaction. Thus, this vast and diverse phytochemical space can theoretically comprise millions of potential metabolites, some of which have a variety of applications [2]. About 2% of these known plants have already been used for medicinal purposes [3].

Several studies have attempted to estimate the size of the chemical space for the plant kingdom, emphasizing the complexity of plant metabolism. These metabolites can be classified into 2 global categories: the primary core metabolites that are broadly shared across all species and secondary metabolites, which are specialized plant compounds. Around 8,000 metabolites that reoccur across multiple species are captured in databases such as PlantCyc [4]. Secondary plant metabolites that have been identified from the literature have been captured in publicly available Natural Product (NP) databases such as COCONUT [5] and LOTUS [6], which cover approximately 125,000 plant-based compounds. These databases provide a foundation for understanding the diversity of plant metabolites, although they represent only a fraction of the vast chemical space yet to be explored.

Early plant metabolomics research estimated that there are 200,000 plant-derived compounds based on the hypothesis that each species produces at least 5 novel secondary metabolites and the assumption that approximately 223,000 plant species were known at the time [7, 8]. It is worth noting that the plant chemical space is a subset of the entire theoretical chemical space, which is much larger and is further reviewed in Supplementary Text 1.

The identification of chemical structures in complex samples, such as plant extracts, has improved with advancements in metabolomics. However, mapping the entire phytochemical space remains a difficult task. Untargeted metabolomics methods, including liquid chromatography–mass spectrometry (LC-MS), still rely on deep sampling across various enrichment and separation techniques. The quickest method for compound annotations is mass spectral library search [9]. Nevertheless, such mass spectral libraries are hampered by the small number of reference mass spectra, specifically in the NP space. A wide array of computational mass spectrometry tools have been developed to support the structure elucidation process [10], including algorithms that utilize fingerprint lookups in databases of known compounds such as CSI:FingerID [11]. The most promising method for identifying metabolites that cannot be found in any database yet is *de novo* machine learning algorithms such as MS2Mol [12]. Nonetheless, structure verification for many novel compounds still requires the help of nuclear magnetic resonance (NMR). Unfortunately, NMR cannot easily be scaled to achieve a similar throughput to LC-MS due to the low sensitivity and very low sample throughput.

In this work, we leverage one of the largest publicly available metabolomics and literature datasets to estimate the chemical space of the plant kingdom. We begin by examining the known chemical space and find that only a few tens of thousands of plant species have been studied, most of them only superficially. The chemical space documented in the literature likely contains several hundred thousand unique structures, providing only a glimpse of the true diversity. We then analyze the overlap between the metabolomics and literature datasets for plants present in both, observing only a moderate alignment. This suggests that a single or a few metabolomics samples per plant cannot capture the full metabolome. Subsequently, we predict the number of unique chemical structures in a metabolomics dataset spanning over 1,000 plant species using various complementary approaches: (i) a formula prediction model, (ii) a *de novo* prediction model, (iii) library-based search combined with the *de novo* model, and (iv) MS2 clustering. Our results indicate that the number of unique structures in this dataset may already exceed current estimates of the phytochemical space. Finally, we project these findings to estimate the total size of the chemical space across the entire plant kingdom, suggesting that it likely spans into the millions. These projections more than likely indicate that over 99% of the phytochemical space remains unexplored, highlighting its vast and largely untapped potential.

## Results

### Exploring the known chemical space of the plant kingdom

**Figure 1:**
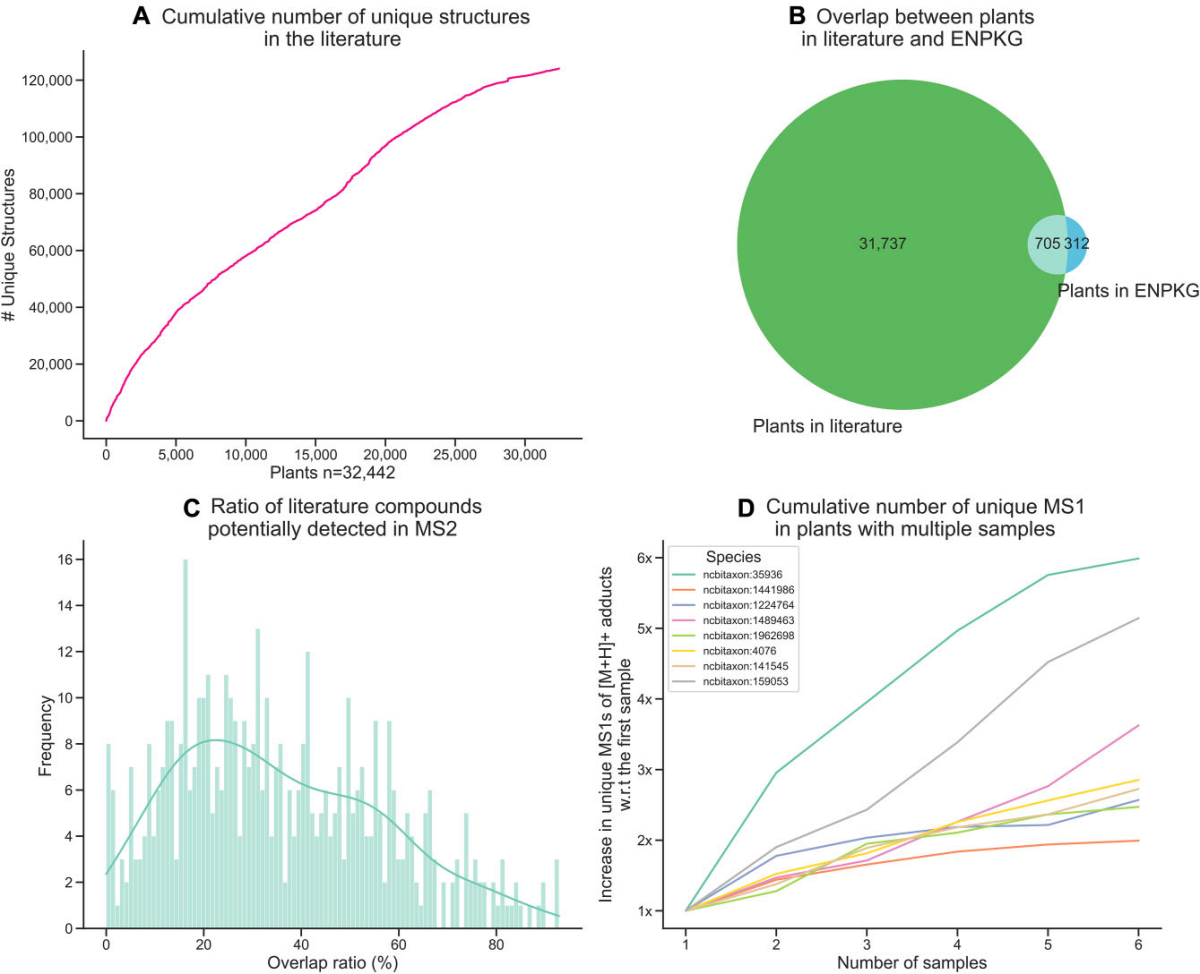
(A) Cumulative curve of unique chemical structures in the literature dataset. (B) Overlap between the plants in the literature and the ENPKG datasets. (C) Distribution of the ratio of literature compounds that can be potentially detected in MS2 spectra based on all the precursor *m/z* mass shifts. Supplementary Table S1 lists the adducts used to calculate each feature’s potential precursor *m/z* mass shifts. (D) Cumulative number of unique precursor masses (MS1) in plants with multiple samples. The y-axis shows the X increase in MS1s as more samples are added. The 8 plants used are the only ones containing more than 5 samples in ENPKG.

Determining the exact size of the chemical space of the plant kingdom remains unattainable with current knowledge. However, we can make a reasonably accurate estimate using publicly available data. To achieve this, we first evaluated the known chemical space of the plant kingdom by constructing a chemical space saturation curve using the 2 largest publicly available literature datasets [5, 6] (Fig. 1A). As expected, the cumulative curve shows a decreasing growth rate as more plants are added, eventually plateauing at approximately 124,000 unique structures for the 32,000 species with available data. Additionally, we examined this trend using Murcko scaffolds and observed a similar pattern (Supplementary Fig. S1). Interestingly, both curves exhibit sharp increases in certain regions, reflecting instances where the addition of a species contributed a disproportionately large number of new structures. This phenomenon highlights biases in the literature, where a few extensively studied species account for thousands of reported structures, whereas most species have only a few, if any, structures documented.

Before estimating the plant chemical space using mass spectrometry data, we first evaluated the coverage of the mass spectrometry dataset in relation to the literature dataset, given their substantial overlap of plants (Fig. 1B). Unfortunately, the literature dataset is sparse, with most plants represented by only a few dozen unique structures (Supplementary Fig. S2). Therefore, we focused our analysis on 490 plants present in both datasets with 20 or more unique structures. As a proxy to assess coverage, we calculated all potential precursor *m/z* mass shifts based on the adducts listed in Supplementary Table S1 and compared them to the exact masses of compounds reported in the literature for the same plant (Fig. 1C). It is important to note that the MS2 dataset does not capture the entirety of the metabolites present in the plants, further constraining the observed overlap. The overlap in Fig. 1B represents the percentage of chemical structures in the literature for which a matching precursor *m/z* mass shift exists, indicating that these compounds could be identified in the metabolomics dataset. Since MS2 signals could correspond to other structures, the observed overlap represents an upper limit of the coverage, as some matches may not directly correspond to the exact compounds reported in the literature. Conversely, the absence of a precursor *m/z* mass shift matching a given compound indicates that the compound is not present in the metabolomics dataset. The overlap ratios across these 490 plants follow a normal distribution centered on 25%, indicating that for most plants, the metabolomics dataset can potentially capture only a moderate proportion of the compounds reported in the literature (Fig. 1C). This confirms that a substantial number of metabolites documented in the literature are not present in the metabolomics dataset.

Another aspect we were interested in investigating is how the coverage of the metabolome increases as more samples are screened for a given species. To do so, we calculated the cumulative number of unique precursor masses (MS1s) rounded to 2 decimals for 8 species containing more than 5 samples in Experimental Natural Products Knowledge Graph (ENPKG) (Fig. 1D). The results show how going from a single sample to 6 increases the metabolome coverage up to 6 times. These findings suggest that while the ENPKG dataset includes over 1,000 plant species, it may capture only a portion of their true chemical diversity. As a result, the estimates derived from this dataset might not entirely reflect the metabolomic richness of these plants, given the current sampling depth.

We also assessed the consistency of metabolite coverage for extracts from 15 plants shared between the Korean Pharmacopoeia [13] and the Pierre Fabre Research Institute Library (RFRIL) [14], the 2 main extract libraries within ENPKG. To do this, we compared the distribution of precursor masses and the overlap of predicted Murcko scaffolds from CSI:FingerID across both libraries (Supplementary Fig. S3). Our analysis revealed noticeable differences in precursor masses and a low overlap in predicted scaffolds between the 2 libraries. Additionally, the RFRIL samples contained a larger number of features compared to the extracts from the Korean Pharmacopoeia. These discrepancies in precursor masses and scaffold overlap likely stem from differences in extraction methods and other factors influencing the metabolites captured in a metabolomics dataset, which are further examined in the Discussion section. Overall, the multiple findings here suggest that the estimates presented in the following subsections likely underestimate the true size of the plant metabolite space.

### Public mass spectrometry data may already surpass current estimates of phytochemical space size

To assess how many unique chemical structures may already exist in current datasets, we leveraged one of the largest publicly available metabolomics datasets for plants (ENPKG) [15]. We used a combination of 4 approaches to get these estimates: (i) predicted formulas (SIRIUS), (ii) *de novo* modeling (MS2Mol), (iii) hybrid (CSI:FingerID + MS2Mol), and (iv) MS2 clustering. We selected MS2Mol since it was one of the first publicly available *de novo* models, and we developed it. We also selected CSI:FingerID because it serves as the standard library reference model. Alternatively, other *de novo* models such as MSNovelist [16] or database lookup approaches (e.g., spectral entropy or cosine similarity) could have been used. These choices allowed us to get varying estimates from 3 widely used approaches with different strengths and limitations.

**Figure 2:**
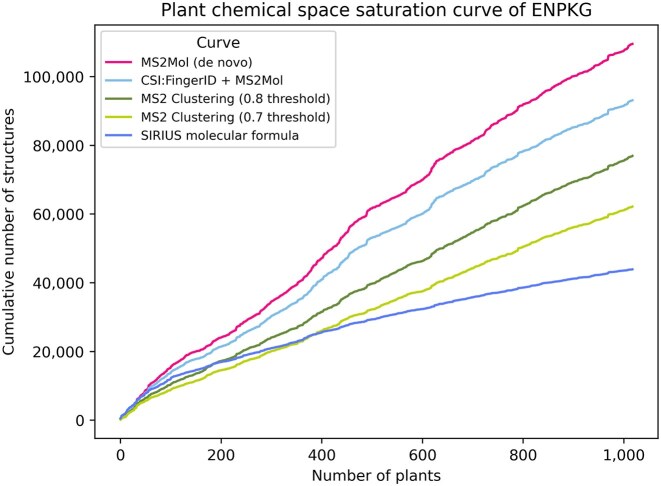
Plant chemical space saturation curve of ENPKG. The plot shows a cumulative curve of the predicted number of unique structures using different methodologies. These curves are used later to fit power law models to estimate the total phytochemical space.

Our results reveal that the *de novo* model predicts the most structures, surpassing 100,000 unique structures across the 1,000 plants. In contrast, the number of unique formulas is the lowest due to its inherent constraint (Fig. 2). MS2 clustering and the hybrid approach (CSI:FingerID + MS2Mol) yield estimates that fall between these 2 extremes. Interestingly, while the formula curve is very likely a substantial underestimate since one formula can correspond to multiple structures, it is higher than the MS2 clustering curve at the beginning. Because the formula curve already provides a conservative baseline, any curve that falls below it is likely underestimating even further. This suggests that the MS2 clustering curves may also be conservative in their estimates. These findings highlight the differing capabilities and scopes of the models. Rather than directly comparing the methods—which is not the focus of this work—we emphasize that the predictions from the *de novo* model, the hybrid approach, and MS2 clustering are close to the size of the literature (124,000 metabolites) with far fewer plants. Overall, given that there are mass spectrometry data for 3,000 plants in Global Natural Products Social Networking (GNPS) and other public repositories and the curves increase linearly, it is more than likely that current mass spectrometry data may exceed current phytochemical space size estimates.

Despite these differences in cumulative estimates, the structure curves are strikingly similar in shape, with steeper increases at consistent points across methods. This indicates that certain plants consistently contribute new chemicals regardless of the approach used. We note that the taxonomic diversity in this public dataset is low compared to a null distribution (Supplementary Table S2 and Supplementary Fig. S4). Thus, we believe a dataset with any randomly drawn 1,000 plants might have shown even a steeper increase.

Additionally, we observed similar trends when analyzing Murcko scaffolds instead of structures (Supplementary Fig. S5). These patterns suggest the models are not simply inflating the numbers based on specific regions of the chemical space but are capturing trends across different plants. To further validate this, we compared the predicted structures for a given MS2 spectrum between the 2 models (Supplementary Fig. S6). We observed a relatively high level of agreement, supporting the consistency and reliability of the predictions.

### Projections of the plant chemical space for plants range from millions to tens of millions

**Figure 3:**
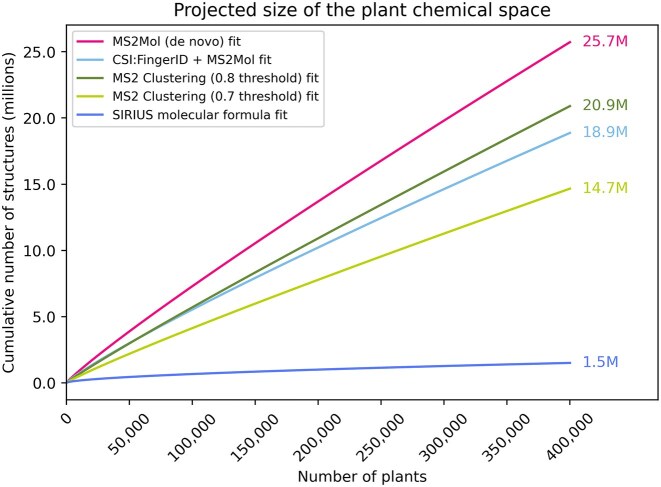
Estimated size of the chemical space after fitting the power law curves across various methodologies. The numbers on the right side correspond to the size of the chemical space in millions for each method, assuming there are 400,000 species of plants. Supplementary Fig. S8 depicts a zoomed-in view of the plot around the first 1,000 and 10,000 species.

To estimate the total number of plant metabolites, we fit power law models to the curves in Fig. 2 and projected them to 400,000 plants, yielding estimates between 1.5 and 25.7 millions total plant metabolites (Fig. 3). The formula curve probably underestimates the size, as a given formula can lead to multiple structures. On the other hand, the MS2Mol curve is affected by the accuracy of the predictions from the model, which was not necessarily trained to get exact matches. Similarly, the hybrid model resembles the MS2Mol curve since most CSI:FingerID predictions have low confidence (Fig. 5A). Given these limitations of the predictive models, the MS2 clustering methods are, in our opinion, the most appropriate for this task.

Additionally, we estimated the number of metabolites for 35,000 plant species, a value comparable to the number of species documented in the literature, to evaluate how our projections align with currently available data. For the MS2Mol model, the predicted number of unique structures approaches 3 million, while MS2 clustering yields estimates ranging from 1.6 to 2.1 million, depending on the similarity thresholds of 0.7 and 0.8, respectively. These findings highlight the significant gap between the predicted metabolite diversity and the literature’s current coverage, emphasizing how much of the plant chemical space remains unexplored.

Lastly, we used holdout sets comprising the last 10%, 20%, and 30% of the data to validate our models. These sets resulted in minor variations in how well the curves fit the data (Supplementary Fig. S7A), magnified when the curves were projected for 400,000 plants (Supplementary Fig. S7B). These variations were most pronounced for the CSI:FingerID curves since they flattened out more quickly than the others. This suggests that as more data become publicly available, these data curves may change and plateau sharply.

In conclusion, our estimates reveal that the lower bound for potential plant metabolites is higher than previously estimated, exceeding earlier estimates by a factor of at least 10. However, the current datasets constrain the upper and lower bounds and could evolve as more public data become available.

## Discussion

Our work relies on multiple factors that can significantly influence the resulting estimates; therefore, discussing the most critical considerations is crucial. Table 1 summarizes the limitations discussed in this section.

**Table 1:** Summary of the major limitations of our work

Limitation	Summary	Effect on estimates
**Dataset coverage**	Estimates are based on a small fraction of plant species, leading to potential biases	Unknown
**Extraction and chromatographic bias**	Different protocols and incomplete anatomical plant coverage may omit specific metabolites	Underestimation
**MS2 Spectrum prediction accuracy**	Library constraints and clustering methods may overestimate or fail to generalize	Unknown
**Disparate solvents used**	Different solvents extract different compounds, causing inconsistencies	Underestimation
**Adducts considered**	Only [M+H]+ adduct was considered	Underestimation
**Missed compound classes**	Polar and moderately polar methods miss volatile and lipid compounds	Underestimation
**False positives in dataset**	Annotation errors and contaminants may inflate metabolite counts	Overestimation
**Functional genomics potential**	Genomics tools could dramatically expand estimations but remain underexplored	Underestimation
**Projection modeling**	Power law projections may overestimate space if actual data curves plateau	Overestimation

First, although we employed one of the largest publicly available datasets, our estimations are based on a metabolomics dataset that represents less than 1% of all species in the plant kingdom. While including the literature dataset expanded the coverage to approximately 10%, only a small fraction of those plants have been studied comprehensively. This limited coverage introduces potential biases, as the plants that have been extensively studied are not necessarily representative of the vast chemical diversity present across the entire plant kingdom. Consequently, our estimates are influenced by the depth and breadth of current data, highlighting the need for further profiling and exploration to better capture the true extent of plant chemical diversity. These limitations are particularly pronounced when projecting curves using power law models to get estimates for 400,000 plants, as the actual data curves may plateau beyond 1,000 plants, leading to lower estimates.

Second, our estimations are derived from metabolomics data obtained through specific extraction methods and chromatographic protocols, which may not capture a substantial portion of the metabolome (discussed later in detail). For instance, the ENPKG data were generated using different extraction techniques and chromatography columns, such as the BEC C18 nonpolar column. Employing alternative protocols could uncover undetected metabolites under the current experimental settings. Additionally, our analysis lacks comprehensive coverage of plant extracts from all anatomical parts—such as roots, stems, and leaves—for most species. This limitation is critical, as metabolite production varies significantly between plant parts and is further influenced by environmental conditions (e.g., season, stress, temperature, humidity) (Fig. 1D). These factors collectively constrain the comprehensiveness of our estimations and suggest that the true diversity of plant metabolites may be even greater than what our analyses indicate (Fig. 1C, D).

For instance, [17] estimated that the size of the chemical space of endophyte-derived secondary metabolites could be in the range of several billion unique compounds. These estimates were derived using assumptions such as ∼90% of secondary metabolite biosynthetic capacity being silent or cryptic, the presence of approximately 10 host-specific bacterial endophytes per plant species, and hundreds of fungal endophyte species per plant. Their approach also relied on rough approximations of species-to-metabolite ratios. While these estimates are impressive, the assumptions may be optimistic since every endophyte species is unlikely to produce entirely distinct metabolites, as overlap and redundancy in biosynthetic pathways are common. Additionally, their calculations assume that each endophyte species acts independently, disregarding interactions between microbes, plants, and environmental factors that may influence metabolite production.

The third major factor is the accuracy of the predicted structures for each MS2 spectrum. Library-based models like CSI:FingerID rely on a fixed library (∼1 million biomolecules), while generative models trained on mass spectrometry data struggle to generalize beyond the chemical space in their training [18]. To address these limitations and focus solely on the number of unique structures in the dataset, we clustered the MS2 spectra to identify groups corresponding to the same chemical structure. This approach, however, is sensitive to the spectral entropy threshold chosen to define the clusters (Supplementary Fig. S9) and may cluster spectra from distinct molecules with similar patterns, especially if they have few peaks. Additionally, differences in the MS/MS acquisition methods—[13] used a collision energy ramp from 20 to 100 eV, while [14] used 3 distinct collision voltage settings (15, 30, and 45 eV)—may lead to overcounting unique structures because they may be represented by slightly different MS/MS fragmentation spectra. To mitigate these issues and avoid overestimation, we made our estimations exclusively relying on the predominant adduct ([M+H]+) and filtered out spectra with fewer than 5 peaks. However, this likely underestimates the true chemical diversity, as other adduct forms, which may represent unique metabolites, are excluded from consideration.

The fourth factor is the disparate solvents used for plant extraction [19]. The Korean Pharmacopoeia dataset [13] used a methanolic extract. Methanol is a polar solvent and extracts a wide range of compound classes (alkaloids, flavonoids, phenolic acids, and tannins). The second dataset [14] utilized ethyl acetate as the extraction solvent, a moderately polar solvent leading to the extraction of sterols, terpenoids, and less tannins. The different solvents used can explain the differences observed among the 15 extracts of plants in both datasets (Supplementary Fig. S3). The fifth factor is that the extraction methods above will not capture many other compound classes and require different measurement technologies. The polar and moderately polar extraction and the LC-MS–based method ignore numerous other substance classes, such as volatile compounds and lipids. For volatile organic compounds (VOCs), gas chromatography coupled to mass spectrometry (GC-MS) has to be used. For polar and neutral lipids, specialized lipidomic extraction techniques have to be applied.

The sixth factor relates to false-positive compounds in the dataset, such as annotation errors from CSI:FingerID and MS2Mol (e.g., plasticizers or other contaminants such as pesticides). While they can be considered correct for sample annotation, they cannot be considered plant metabolites [20]. For example, we have observed several thousand fluorinated and chlorinated SMILES structures in annotations from both models. To confirm the presence or absence of such compounds, one would need to run additional analytical tests, which go beyond the scope of this article. The seventh factor is a further path that relies on functional genomics approaches by massively increasing the compound space for compounds of interest. That can be done by CRISPR/Cas-based gene editing and other advanced techniques, including cytochrome P450 transform in plants [21].

In summary, while our study provides a foundational estimate of the size of the plant chemical space, significant opportunities remain for future research to refine and improve these projections. The limitations listed in Table 1 highlight the need for broader datasets and experimental approaches to capture more of the plant metabolome space. As more data become available through sources like MetaboLights [22], Metabolomics Workbench [23], and GNPS [24], the curves can be regenerated and the estimates adjusted accordingly.

## Potential Implications

In this study, we sought to estimate the size of the chemical space of plants by leveraging one of the largest publicly available metabolomics and literature datasets. While deriving an exact number remains infeasible, we employed multiple approaches to establish a robust estimate of the potential range of the plant chemical space under various assumptions. First, we examined the known chemical space by analyzing the saturation curve derived from 124,000 unique chemical structures documented across over 30,000 plant species in the literature. Our findings highlight that the chemical space exhibits signs of saturation as additional species are studied, reflecting most plants’ limited exploration depth.

Next, we extended our analysis to metabolomics datasets comprising over 1,000 plant species. By leveraging mass spectrometry data, we provided a broader perspective on how the chemical space expands with increased profiling. Notably, the estimates for the number of unique structures across the 1,000 plants analyzed are already close to previous estimates of the chemical space [7, 8] and the number of structures documented in the literature. Furthermore, after we modeled growth curves to extrapolate the size of the chemical space based on an estimated 400,000 plant species, even the most conservative estimates, using formula prediction, reached into the millions. Based on molecular formulas, we estimated 1.5 million unique compounds, which, while relatively reliable, almost surely underestimate the true diversity. Additionally, the different structure prediction and MS2 clustering approaches estimated at least 15 million unique structures. Collectively, these findings suggest that plants’ chemical space could range from several million to tens of millions of unique structures.

## Methods

### Collecting publicly available mass spectrometry data from plants

We identified several of the largest datasets publicly available through the ENPKG, a Knowledge Graph structured in a Resource Description Framework (RDF) to harmonize heterogeneous NP metabolomic datasets [15]. Apart from harmonizing several datasets and allowing query them, ENPKG contains thousands of associations between LC-MS features and their corresponding metadata, such as their SIRIUS/CSI:FingerID-predicted structures [11, 25], references to the ISDB-LOTUS database [26], and references to their derived extracts. These metadata are externally linked using standard identifiers such as WikiData for extract species, chemical class through NPClassifier [27], and predicted structure through SMILES [28], InChIKeys, and ChEMBL identifiers [29]. Throughout the article, we refer to chemical structures as plant metabolites interchangeably. The current version of ENPKG integrates 3 medicinal plant datasets and 3 bacterial datasets (*Leishmania donovani, Trypanosoma cruzi*, and *Trypanosoma brucei rhodesiense*). We restricted our analysis to the 3 medicinal plant datasets (see details in Table 2).

**Table 2:** Overview of the 3 medicinal plant datasets in ENPKG. The first column references the MassIVE ID of the dataset, where the metabolite spectra can be found. The second and third columns are the plant library and its original publication. The last 3 columns report the number of extracts in the library, the number of unique structures (represented by SMILES) predicted by CSI:FingerID, and the number of features detected. Note that the number of extracts does not correspond to the number of plant species, as the datasets could have multiple extracts for 1 species

MassIVE ID	Plant library	Reference	# Extracts	# Unique structures	# Features
MSV000093464	Korean Pharmacopoeia Plants Extracts	[13]	337	17,428	42,520
MSV000088521	*Waltheria Indica* Samples (aerial parts and roots)	[30, 31]	1	2,453	4,422
MSV000087728	Pierre Fabre Research Institute Library (RFRIL)	[14]	1,600	65,381	784,836

We queried the ENPKG (accessed on 26 November 2024) via their SPARQL endpoint in GraphDB [32] to extract all LC-MS features with the following annotations: (i) an InChIKey; (ii) a SMILES; (iii) NP class, superclass, and pathway annotation from NPClassifier; (iv) structural annotation from CSI:FingerID and confidence scores; (v) predicted formula from SIRUS; and (vi) a WikiData species annotation. The SPARQL query used for this extraction is provided in Supplementary Text 2. This resulted in 76,982 unique chemical structures and 831,778 features across 1,018 unique plant species (Fig. 1A). We would like to note that we used a consistent arbitrary ordering of these plants in our analyses, but we confirmed that the ordering does not significantly affect the results (Supplementary Fig. S10). Finally, we downloaded the raw spectra for these features using MassIVE’s API [33].

The 831,778 features in the dataset correspond to various adduct types (Supplementary Table S3). To reduce potential redundancy, we filtered out all features not predicted as [M+H]+ adducts by SIRIUS [25], resulting in 335,377 features. While this step excludes over 50% of the features and disregards negative mode samples, it significantly reduces the risk of overestimating the projections for the chemical space. Notably, we did not apply this filtering step to the formula prediction method since it accounts for adducts.

We further categorized the predicted structures into known and unknown groups based on an arbitrary CSI:FingerID confidence score threshold of 0.5. These confidence scores, generated using COSMIC [34], have been proven to separate correct and incorrect structures (using the CASMI 2016 dataset). About 75% of the features from the dataset were classified as unknown (34,808 chemical structures), while the remaining 25% were categorized as known (12,434 chemical structures). The rationale behind this classification was to annotate later unknown features (i.e., low confidence annotation), which are likely not in the reference library, using a *de novo* structured prediction model. Fig. 5 details the confidence scores, each group’s features percentage, and MS precursor mass distribution. Supplementary Figs. S2 and S3 also show the distribution of the predicted NP super class and classes using CANOPUS [35].

Lastly, we note that the quality of the public datasets used constrains the methods described below for estimating the size of chemical space. Notably, we found that 1.04% of the spectra in the ENPKG dataset had a dominating peak with more than 90% of the total intensity. Such spectra are generally uninformative and may not have been accurately classified by predictive models or MS2 clustering methods. To help mitigate this issue, we removed any spectra from the dataset with fewer than 5 peaks before creating the data curves.

### Harnessing chemical structures reported in plants across the scientific literature

**Figure 4:**
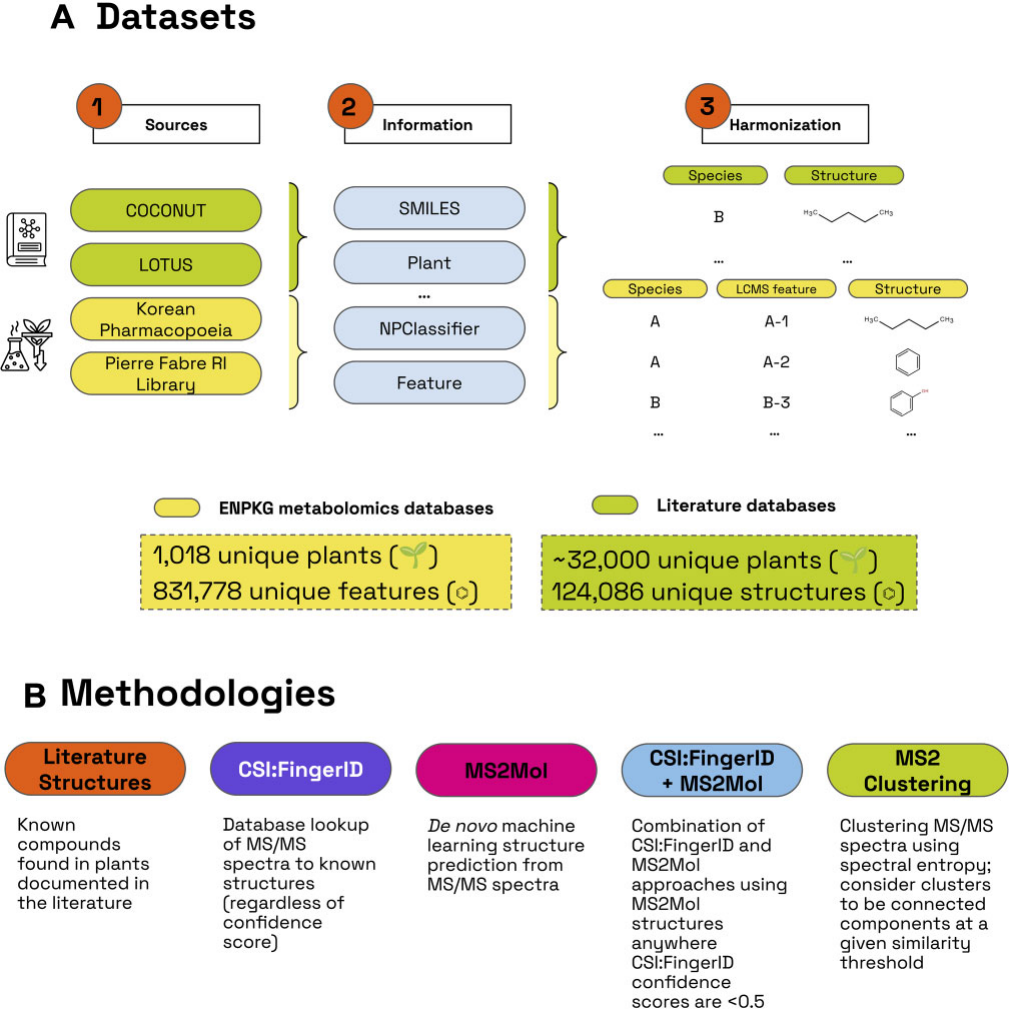
(A) Datasets leveraged in our work and the corresponding information extracted from them. (B) Methodologies employed to calculate the number of unique chemical structures in the datasets.

While the mass spectrometry datasets described in the previous section provide a snapshot of the metabolites present in a specific plant under specific conditions, the LC-MS features identified for a given plant are far from representing all actual metabolites present in the sample. Without going deep into the topic, which is mentioned later in the Discussion, various technical factors affect the detected chemical space of a given sample, such as chromatography column and extraction method. Furthermore, different plant parts will produce distinct metabolites, and the abundance of a metabolite in the mixture can be, in some cases, too low to be detected. Thus, we also complement our analysis with 2 of the largest curated datasets comprising metabolites that have been reported in scientific literature to be in the natural world: COCONUT 2.0 [5] and LOTUS [6] (Fig. 4A).

**Figure 5:**
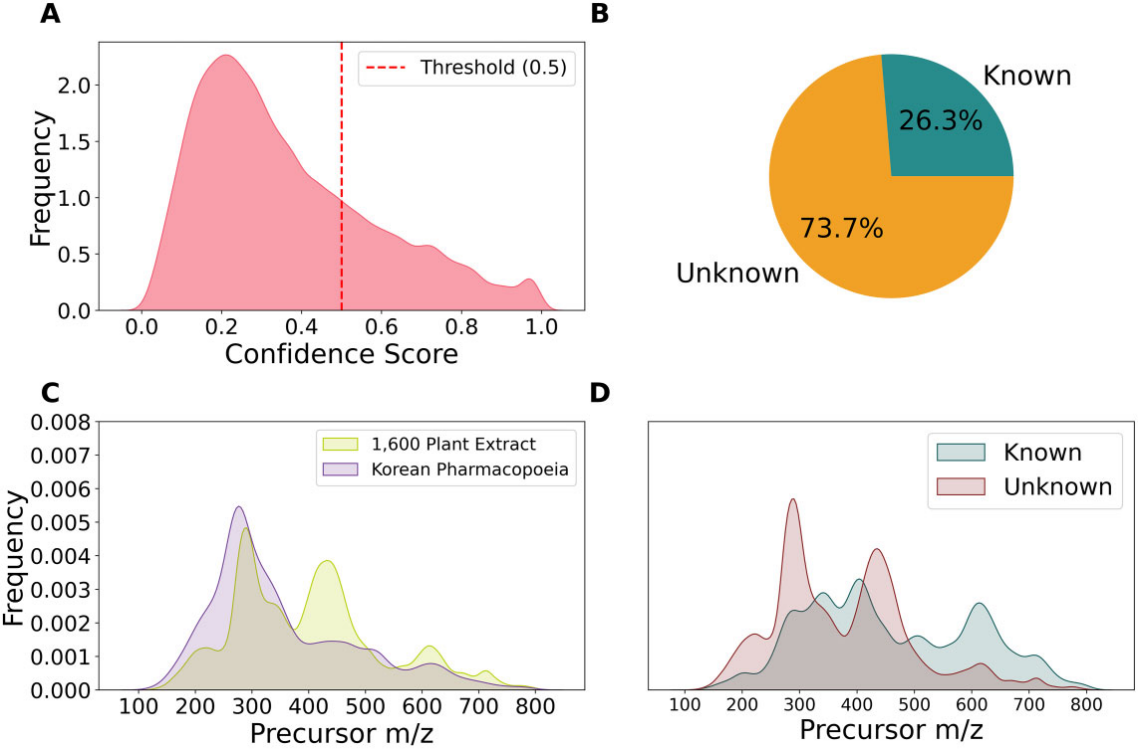
Summary of the ENPKG dataset. (A) Distribution of the CSI:FingerID confidence scores for the predicted structures across all features with a predefined threshold of 0.5 (dotted red line) to classify confidently predicted (known) and unknown features. (B) Distribution of confidently predicted (known) and unknown features in the dataset. (C) Distribution of the precursor *m/z* across all the features stratified by the 2 primary datasets. (D) Distribution of the precursor *m/z* for known and unknown features in the dataset.

While both datasets comprise multiple sources of information, given our goal, we simplified the datasets to the associations between chemical structures and their reported taxonomic species. The datasets capture this information in 2 columns: SMILES and species name. However, given that both datasets contain other taxonomic clades apart from plants, we assigned all taxonomic species to a unique identifier of the following plant taxonomic nomenclatures: NCBITaxonomy [36], Integrated Taxonomic Information System (ITIS), and World Flora Online (WFO). For NCBITaxonomy, we subset exclusively to all species under the Viridiplantae kingdom (NCBITaxon:33090).

To avoid the indistinctive assignment of species to different nomenclatures, we established a prioritization order, namely: (i) NCBITaxon, (ii) ITIS, and (iii) WFO. Thus, if a species name is present in several nomenclatures, it is exclusively assigned to the identifier of the nomenclature with the highest priority. After applying this harmonization approach, this prioritization is reflected in the final distribution of plants captured in both datasets (Supplementary Table S4). Lastly, we parsed SMILES with RDKit [37] and converted them to InChIKeys (first 14 characters) while filtering out invalid SMILES. The number of unique structures identified was 124,086.

### Predicting formulas and structures from MS2 data

We used 3 methods to calculate the unique number of structures and generate structure curves from MS2 data (Fig. 4B). First, we also employed a *de novo* model, MS2Mol [12], which can predict novel structures in previously unexplored regions of chemical space. Second, we used a hybrid approach combining the confident predictions from CSI:FingerID (i.e., known group) with the MS2Mol predictions for the remaining spectra. Third, we predicted the formulas (which can lead to multiple structures) for each feature to establish a lower bound using SIRIUS, a state-of-the-art formula prediction model [25], acknowledging that each formula corresponds to multiple possible structures. Note that because we do not use a similarity threshold for the MS2Mol model, it may provide an overestimate of the number of novel structures. While introducing such a threshold from a trained confidence model would decrease the number of predicted structures, it could also lead to an underestimation since some features might not receive any prediction. We relied on the formula curve for an underestimate and thus allowed the MS2Mol curve to potentially overestimate the number of predicted structures, providing us with a valuable range of estimates.

### Spectral clustering

As an alternative method for estimating the number of structures, we implemented MS2 clustering to avoid dependence on predictive models (Fig. 4B). Using the spectral entropy metric [38], we constructed a similarity matrix for the spectra in our dataset. We limited comparisons to spectra with precursor *m/z* within 10 and 200 ppm to minimize false matches, assigning a similarity score of zero to all other spectral pairs. While the results in this article are based on a 10-ppm filter, no significant changes were observed when using a 200-ppm filter. Clustering was performed by thresholding the similarity scores at 0.5, 0.6, 0.7, 0.8, and 0.9 (Supplementary Fig. S9). The article primarily relies on results using thresholds of 0.7 and 0.8, aligning closely with the 0.75 threshold reported by [38] to have a false discovery rate of less than 10%. Finally, we constructed a network with *igraph* [39] and identified clusters based on the connected components.

### Projecting the total chemical space

Applying the previously described estimation methods to the public ENPKG dataset gave us estimates for the number of plant metabolites in 1,000 plants. To project the total number of plant metabolites across all 400,000 plant species, we fitted power law models (Supplementary Function 1) to the data. We employed holdout sets of 30%, 20%, and 10% to validate our predicted curves, observing minor variations (Supplementary Fig. S7).

### Implementation

All scripts used in this work were written in Python version 3.10 and managed using the Poetry environment [40]. For data manipulation, we employed widely used libraries such as Pandas [41], NumPy [42], and SciPy [43]. Visualizations were generated using seaborn [44] and Matplotlib [45]. To represent chemical structures, we first employed SMILES that were converted to InChIKeys using RDKit [37]. Notably, we represented chemical structures using the first block of 14 characters from the InChIKey (out of a total of 27) to intentionally exclude stereochemistry, since considering it would significantly inflate estimations. For MS2 data processing, we relied on specialized libraries, including MatchMS [46]. The clustering of MS2 data was performed using the *igraph* library [39].

## Availability of Source Code and Requirements

Project name: Chemical Space Estimation

Project homepage: https://github.com/enveda/chemical-space-estimation/ [47]

Operating system(s): Linux, MacOS, Windows

Programming language: Python

License: GNU General Public License v3.0

RRID:SCR_026498

## Supplementary Material

giaf033_Supplemental_File

giaf033_GIGA-D-25-00011_Original_Submission

giaf033_GIGA-D-25-00011_Revision_1

giaf033_GIGA-D-25-00011_Revision_2

giaf033_Response_to_Reviewer_Comments_Original_Submission

giaf033_Response_to_Reviewer_Comments_Revision_1

giaf033_Reviewer_1_Report_Original_Submission Carlos RodrÃguez-LÃ^3^pez -- 2/19/2025

giaf033_Reviewer_2_Report_Original_Submission Kohulan Rajan -- 2/24/2025

## Data Availability

A snapshot of our code in GitHub has also been archived in Software Heritage [48]. All datasets supporting this work are publicly available and can be directly downloaded from Zenodo [49].

## Additional Files

**Supplementary Text 1**. Literature review on estimating the entire size of the chemical space.

**Supplementary Text 2**. SPARQL Query to extract data from ENPKG.

**Supplementary Fig. S1**. Cumulative curve of unique Murcko scaffolds in the literature dataset. In total, there are 34,371 unique Murcko scaffolds in the literature datasets. Given that there are 124,086 unique structures, there is an average new Murcko scaffold for every 3.61 novel structures.

**Supplementary Fig. S2**. Distribution of unique chemical structures in the literature dataset. Most of the plants exclusively contain a few structures.

**Supplementary Fig. S3**. Distribution of precursor masses and overlap of Murcko scaffolds predicted with CSI:FingerID for the 15 plants present in both the Korean Pharmacopoeia dataset [13] and the Pierre Fabre Research Institute Library (1,600 plant extracts) [14] datasets. We observe significant differences in the precursor masses distribution between the 2 datasets for the same plant and on the predicted Murcko scaffolds. Additionally, for these 15 plants present in both datasets, the extracts from the 1,600 plant extract dataset contain more features than those from the Korean Pharmacopoeia dataset. As discussed in the introduction, these differences are probably due to the different extraction methods used, among other factors. The high-quality version of this figure is present in the GitHub repository, as we could not upload it, retaining the original quality here.

**Supplementary Fig. S4**. Evaluating the diversity of ENPKG compared to a null distribution. The red line represents how many orders, families, and genera are in the ENPKG dataset. We compared these numbers to a null distribution of orders, families, and genera in 10,000 random samples of the same size (same number of species) as the ENPKG dataset. The bottom line is that the ENPKG dataset has a lower diversity of orders, families, and genera compared to what you would expect by chance if you pick random species from the plant kingdom of the same size as the ENPKG dataset (∼1,000 species). This is because the ENPKG dataset focuses on specific genera/families, like the ones from Korea, making it less diverse than a completely randomly distributed sample.

**Supplementary Fig. S5**. Plant chemical space saturation curve of ENPKG using Murcko scaffolds of the predicted structures from MS2Mol.

**Supplementary Fig. S6**. Distribution of Tanimoto similarity between the chemical structures predicted with MS2Mol and CSI:FingerID. The Tanimoto similarity is calculated for each pair of predicted SMILES for a given MS2 using RDKit fingerprints with default parameters. The average Tanimoto coefficient is 0.51, the standard deviation is 0.28, the 25th percentile is 0.29, the 50th percentile is 0.45, and the 75th percentile is 0.70.

**Supplementary Fig. S7**. Comparison of training ratios for the power law model. (A) Curves fitted using different training ratios (0.7, 0.8, 0.9, 1) compared to the actual curve of the predicted formulas in the approximately 1,000 plants in the metabolomics dataset. (B) Projection of the curves fitted in (A) using different training ratios on the 400,000 species. Note that while the fitting ratio seems similar in the first 1,000 plants, these subtle differences translate into differences of several hundred thousand unique compounds when extrapolating to all 400,000 species.

**Supplementary Fig. S8**. The projected size of the plant chemical space after fitting power law models on the different methodologies on the metabolomics dataset. (A) This plot is a zoomed-in version of Fig. 4 in the article. We can see how the fitted curves for the projections fit the actual curves from the metabolomics datasets. The different assumptions/nuances across the methods explain the differences in the curve shape. (B) When zoomed in to 10,000 plants, we can see that the CSI:FingerID + MS2Mol curve eventually crosses the MS2 Clustering (0.8 threshold) curve as the hybrid curve plateaus more significantly. This behavior is likely due to the influence of CSI:FingerID predictions, which are constrained by a reference library.

**Supplementary Fig. S9**. Various spectral entropy thresholds were employed for MS2 clustering, resulting in projections for the number of metabolites in all plant space ranging from 6.8 to 34.8 million. For the main results of our article, we use the moderate estimates.

**Supplementary Fig. S10**. Shuffling the order of the plants does not significantly change the shape of the curve. Because the curves in Fig. 3 all have similar shapes, the plant ordering differences will be similar for the other datasets. Each color represents a curve made in a different order. The curves look similar regardless of the methodology used (e.g., formulas from SIRIUS, MS2Mol predictions). While minor variations in curve shape caused by shuffling the order may carry over to the projected curves in Fig. 5, these differences will not meaningfully impact the range of the estimates, which is the primary focus of that figure.

**Supplementary Table S1**. List of adducts used to calculate the potential precursor *m/z* mass shifts that are later matched against the exact masses of reported compounds in the literature for a given plant.

**Supplementary Table S2**. Number of taxonomic clades covered by the metabolomics datasets. In total, the dataset covers 672 genera, which means, on average, there is approximately 1 new genus for every 6 species. Similarly, there are a total of 181 families, which corresponds to approximately 5 species per family. The most common family is *Fabaceae*, which has 77 plants (7.5% of the total species). The most common genus is *Garcinia*, which has 9 plants (0.88% of the total species).

**Supplementary Table S3**. Distribution of each adduct predicted by SIRIUS for all 831,778 features in ENPKG.

**Supplementary Table S4**. Number of unique plants in the combined literature dataset (COCONUT and LOTUS) after species harmonization.

**Supplementary Function 1**. Power law model used to project plant metabolite count curves.

## Abbreviations

CASMI: Critical Assessment of Small Molecule Identification; COCONUT: COlleCtion of Open Natural prodUcTs; COSMIC: Confidence Of Small Molecule IdentifiCations; CRISPR: clustered regularly interspaced short palindromic repeats; ENPKG: Experimental Natural Products Knowledge Graph; GC-MS: gas chromatography coupled to mass spectrometry; GNPS: Global Natural Products Social Networking; ITIS: Integrated Taxonomic Information System; LC-MS: liquid chromatography–mass spectrometry; MS/MS: tandem mass spectrometry; NP: natural product; NMR: nuclear magnetic resonance; RDF: Resource Description Framework; RFRIL: Pierre Fabre Research Institute Library; VOC: volatile organic compounds; WFO: World Flora Online.
