# Supplementary material for: Defining the limits of plant chemical space: challenges and estimations
Source: Gigascience. 2025 Apr 4;14:giaf033. doi: 10.1093/gigascience/giaf033 (PMC11970369; doi:10.1093/gigascience/giaf033)
Supplement: giaf033_Response_to_Reviewer_Comments_Original_Submission [file giaf033_response_to_reviewer_comments_original_submission.pdf]

Author's Response To Reviewer Comments

We believe the current version of the manuscript addresses all comments raised by the reviewers. We have replied one by one to each comment below, starting with a double asterisk (**).

Reviewer reports:

Reviewer #1: In the reviewed manuscript, Chloe Engler Hart et al. utilize different approaches to estimate the size of plant chemical space through analysis of publicly available datasets of mass spectrometry-based metabolomics. The authors tackle this issue by using data from ca. 2,000 LC-MS runs, and different formula predictors and structure annotation algorithms, and extrapolate to the estimated number of plant species. While the approach is useful at estimating structural variation, and the collected data and here-published source code can certainly be of use to the plant metabolomics community, I consider the manuscript requires modifications before it can be recommended for publication.

** We would like to thank the reviewer for the thoughtful and positive feedback. We believe that we have addressed your comments below. Our replies start with double asterisks (**).

> Particularly, the language of the article should more accurately reflect the nature of this estimate; for example, mentions of the approach being "the most accurate estimate possible" (p.8, section 3.2) are not supported, and throughout the article, mentions of the calculation as a "conservative estimate" are not consistent with the approaches used, beyond formula prediction. E.g. it is mentioned that the MS2 curve being lower than formula prediction suggests that the curves may be conservative without further clarification on why this might be the case and not, e.g., a product of estimates dispersion. The authors mention that since they identify most limitations (in table 2, p. 13) are underestimations (again, with limited or no explanation) their estimate is conservative. Since no effect size can be calculated on these limitations, this statement is not true; e.g. if the approach is missing half of molecules due to extraction, and another half due to tissue coverage (total, ¼), but overestimating the plateau of plant chemical diversity by 100-fold, even if more factors underestimate the chemical space, the effect size of the latter would be dominant by far.

** Thank you for your feedback. We have adjusted the language throughout the article regarding the estimates being conservative. Specifically, we revised the sentence in section 3.1 that you highlighted, clarified our reasoning for suggesting that the MS2 curve might be an underestimate in section 3.2, and removed references to the estimates being overall conservative from the discussion and conclusion.

> I recommend the authors to change mentions of this estimate being a conservative approach, and instead clearly mention that this is a fragmentation-based estimate, or a similar term that better reflects the nature of the figure.
Similarly, assumptions on the models should be explicitly stated, along with their limitations. The authors, for example, rely on CID induced fragmentation, and they mention that the estimate "[relies] on the predominant adduct ([M+H]+)" (p.15) and thus "this likely underestimates the true chemical diversity, as other adduct forms" (p.15). It should be stated that this is an assumption: the authors do not have evidence for the adducts being [M+H]+, which is nigh impossible with the available data, they are assuming all features are [M+H]+ adducts. This carries the implicit assumption that fragmentation mechanisms will be the same for all MS2 spectra and thus structural diversity can be estimated through MS2 clusters. It is unclear how this would yield an underestimation, as the authors claim, but rather yields an overestimation, as fragmentation of [M+H]+ and e.g. [M+Na]+ adducts of the same molecule would yield different fragmentation patterns, given the former favors charge migration dependent mechanisms compared to the latter. Thus, since the authors consider all features to be [M+H]+, two adducts of the same molecule might be considered as different moieties, given that fragmentation patterns will differ, even if no difference exists.

** We believe there is a misunderstanding here as we have used the predicted adducts, and we have not assumed that all features are [M+H]+ (as detailed in the 3rd paragraph of Methods section 2.1, and adduct breakdown in Supplementary Table 1). Consequently, we reduced the dataset to 40.3% of the total features to avoid the specific issue that the reviewer points out. Considering all adducts gave us much larger estimates (71 millions for MS2Mol, 67.3M for 0.7 MS clustering, 45.8M for 0.8 clustering, and 55.7M for CSI:FingerID + MS2Mol fit), which did not present in the paper for the same reason the reviewer points out. The submission system does not allow us to attach the figure with these estimates using all adducts, unfortunately.

> On the same vein, since similarity thresholds of the MS2Mol algorithm are essential for the estimation of diversity, the authors should clearly state how are they calculated in text, not by reference, along with potential limitations.

** In this work, we decided not to train a confidence model for MS2Mol or implement a similarity/confidence threshold. This means that we get an MS2Mol prediction for each feature regardless of whether the model is confident or not. While we acknowledge that this decision may lead to the MS2Mol curves being an overestimate, adding a threshold could instead cause an underestimation by leaving some features without predictions. By using the formula curve as a conservative lower bound and allowing the MS2Mol curve to potentially overestimate the number of predicted structures, we achieve a valuable range of estimates. We have added some clarification to the end of section 2.3 to explain this point.

> Finally, I believe the work would greatly benefit from including data on phylogenetics of the samples, adding diversity estimates to their sample and extrapolation data. If, for example, most of the 400,000 plant species are phylogenetically distant from the sampled species, then the reader can reasonably assume that this might be an underestimation of chemical diversity when presented with the evidence. If, on the other hand, the original sample has more diversity than the total number of plant species, this might not be the case. In any case, all of the relevant assumptions should be clearly stated.

** Thank you for the suggestion. We have added a phylogenetic analysis and compared the number of unique genera, families, and orders in our sample against a null distribution drawn from 10,000 random samples of species drawn from the plant taxonomy (see notebook for the analysis / Supplementary Figure 8). The results show that the ENPKG dataset has a lower diversity of orders, families and genus, compared to what you would expect by chance if you pick random species from the plant kingdom of the same size as the ENPKG dataset (~1,000 species). This is because the ENPKG dataset focuses on specific genera/families, (e.g., for example the Korean part of the dataset focuses on certain familiar/genera autoctonous from Korean), making it less diverse than a completely randomly distributed sample. We have now added these findings to the Results section.

> Minor note: One of the main arguments for extrapolating the diversity estimate into the rest of the plants comes from Figure 3D, where increasing MS1 adducts increases with number of samples; it would greatly help explaining the difference seen between species if the authors clarify the tissues sampled per species. E.g. if the species that only doubles the number of features contains only aerial and vegetative tissue, compared to the species that increases 6 fold which might include root or reproductive tissue, etc. This might also help the authors in justifying the extrapolation of the estimate.

** Thank you for the suggestion; this is indeed an interesting thing to explore that could explain the results in Figure 3D. Interestingly, ncbitaxon:35936 / Lablab purpureus, the plant with the 6x increase, is the only one with more than five samples present in both datasets (Korean and RFRIL). As mentioned in the paper, both datasets differed in the extraction method and chromatographic column, so this could explain why this plant has exhibited this trend (6x) and the rest exhibited ~2-3x. However, the extracts of these plants are derived from a minimum of three different parts, up to five. Since there is not a lot of variability, and they all come from the same plant parts, and the sample size is just eight plants, we do not think it is appropriate to derive conclusions from it. Thus, we decided not to include this follow-up analysis in the manuscript.

Reviewer #2: Review: Defining the limits of plant chemical space: challenges and estimations

> This work presents an important contribution to understanding the chemical diversity of plants through a systematic analysis combining metabolomics data and literature mining. The authors address a question in the field and employs multiple complementary approaches to estimate the size of the plant chemical space.

** We would like to thank the reviewer for the thoughtful and positive feedback. Our replies start with double asterisks (**).

> Here are my few suggestions and question to the authors to clarify,

> 1. When introducing an abbreviation one could use caption letters "Natural Products (NP)"

** Thank you for the suggestions. We have now checked all abbreviations and are now introduced with caption letters.

> 2. There is no list of abbreviations in the document, so introduce them first and then use them. There may be some readers who are unfamiliar with the terms COCONUT and LOTUS.

** We have now added an abbreviation section at the bottom of the document, following GigaScience's guidelines (it has to be at the end).

> 3. Is there any prior work using similar combined metabolomics/literature approaches to estimate plant chemical space? If so, these should be cited. If not, please state this explicitly to highlight the novelty of your method.

** We believe that we have already covered all related work in the introduction. Additionally, we have a Supplementary Text outlining related work to estimate the entire chemical space. In the last paragraph of the introduction, we highlight the novelty of our method.

> 4. Cite SMILES

** We have now added this citation.

> 5. While the paper describes the use of 'literature datasets,' it appears that only existing databases (COCONUT and LOTUS) are being utilized. It would be helpful if authors could clarify whether any direct literature mining was conducted. If not, consider revising terminology to more accurately reflect the use of curated databases rather than primary literature sources.

** Thanks for the suggestion. We have now clarified this in methods by adding "curated databases". We do not think it is necessary to change the term "literature datasets" in the abstract and other places because "curated databases" do not clarify where the information comes from ("the scientific literature"). Additionally, being fully explicit with terms like "curated literature-derived chemical repositories" makes the sentences too long and confusing. We hope the reviewer agrees with the current changes, but we are happy to change it if the reviewer has another suggestion.

> 6. Great to see the data and code openly shared on both Zenodo and GitHub. I also find the GitHub repository very useful with regard to all the provided notebooks. To maximize reusability, please consider adding a detailed "How to Use" section to the README that guides others in replicating or building upon this work.

*We have now added a "How to use" section to the README in GitHub (https://github.com/enveda/chemical-space-estimation/blob/main/README.md) and extended the repository's current documentation.

> 7. The different clustering thresholds (0.7 vs 0.8) lead to notably different estimates. Could you discuss which threshold might be more appropriate for this specific application to plant metabolomics data?

** Given the goal of this manuscript (i.e., estimating the chemical space of plants), we believe that providing two estimates (using thresholds 0.7 and 0.8) is more robust than selecting one threshold. Given that all our methods have limitations (as detailed in Table 2), presenting a range of estimates offers more insight than one fixed value. We picked the thresholds of 0.7 and 0.8 because they are close to the threshold of 0.75 reported in the spectral entropy paper (https://doi.org/10.1038/s41592-021-01331-z) as having a false discovery rate of less than 10% (see Methods). While we could add an additional curve for a threshold of 0.75 in Figures 4 and 5, we feel that doing so might clutter the graphs and compromise their readability, especially since the 0.7 and 0.8 curves already provide a useful estimated range. However, we are happy to make this change if the reviewer would prefer it.
