# Supplementary material for: Defining the limits of plant chemical space: challenges and estimations
Source: Gigascience. 2025 Apr 4;14:giaf033. doi: 10.1093/gigascience/giaf033 (PMC11970369; doi:10.1093/gigascience/giaf033)
Supplement: giaf033_Response_to_Reviewer_Comments_Revision_1 [file giaf033_response_to_reviewer_comments_revision_1.pdf]

Author's Response To Reviewer Comments

We have formatted the manuscript accordingly as asked by the production team.

We have come across "Research" publications published in GigaScience (e.g., https://doi.org/10.1093/gigascience/giaf006) that do not include a "Data Description" section. This is the reason why we did not add this section to our manuscript. Given that we have already provided extensive information on Zenodo (https://zenodo.org/records/14940366), we were wondering whether this section is mandatory for our manuscript. Again, we are happy to add it but it is going to be a bit redundant with the other sections and since our manuscript is not focused on a Dataset but on the analysis of the dataset, we do not think it requires such a section.
