# Supplementary material for: Defining the limits of plant chemical space: challenges and estimations
Source: Gigascience. 2025 Apr 4;14:giaf033. doi: 10.1093/gigascience/giaf033 (PMC11970369; doi:10.1093/gigascience/giaf033)
Supplement: giaf033_Reviewer_1_Report_Original_Submission — Carlos RodrÃguez-LÃ3pez -- 2/19/2025 [file giaf033_reviewer_1_report_original_submission.pdf]

Reviewer Report

Title: Defining the limits of plant chemical space: challenges and estimations

Version: Original Submission **Date:** 2/19/2025

Reviewer name: Carlos RodrÃguez-LÃ³pez

Reviewer Comments to Author:

In the reviewed manuscript, Chloe Engler Hart et al. utilize different approaches to estimate the size of plant chemical space through analysis of publicly available datasets of mass spectrometry-based metabolomics. The authors tackle this issue by using data from ca. 2,000 LC-MS runs, and different formula predictors and structure annotation algorithms, and extrapolate to the estimated number of plant species. While the approach is useful at estimating structural variation, and the collected data and here-published source code can certainly be of use to the plant metabolomics community, I consider the manuscript requires modifications before it can be recommended for publication.

Particularly, the language of the article should more accurately reflect the nature of this estimate; for example, mentions of the approach being "the most accurate estimate possible" (p.8, section 3.2) are not supported, and throughout the article, mentions of the calculation as a "conservative estimate" are not consistent with the approaches used, beyond formula prediction. E.g. it is mentioned that the MS2 curve being lower than formula prediction suggests that the curves may be conservative without further clarification on why this might be the case and not, e.g., a product of estimates dispersion. The authors mention that since they identify most limitations (in table 2, p. 13) are underestimations (again, with limited or no explanation) their estimate is conservative. Since no effect size can be calculated on these limitations, this statement is not true; e.g. if the approach is missing half of molecules due to extraction, and another half due to tissue coverage (total, Â¼), but overestimating the plateau of plant chemical diversity by 100-fold, even if more factors underestimate the chemical space, the effect size of the latter would be dominant by far.

I recommend the authors to change mentions of this estimate being a conservative approach, and instead clearly mention that this is a fragmentation-based estimate, or a similar term that better reflects the nature of the figure.

Similarly, assumptions on the models should be explicitly stated, along with their limitations. The authors, for example, rely on CID induced fragmentation, and they mention that the estimate "[relies] on the predominant adduct ([M+H]+)" (p.15) and thus "this likely underestimates the true chemical diversity, as other adduct forms" (p.15). It should be stated that this is an assumption: the authors do not have evidence for the adducts being [M+H]+, which is nigh impossible with the available data, they are assuming all features are [M+H]+ adducts. This carries the implicit assumption that fragmentation mechanisms will be the same for all MS2 spectra and thus structural diversity can be estimated through MS2 clusters. It is unclear how this would yield an underestimation, as the authors claim, but rather yields an overestimation, as fragmentation of [M+H]+ and e.g. [M+Na]+ adducts of the same molecule would yield different fragmentation patterns, given the former favors charge migration dependent mechanisms compared to the latter. Thus, since the authors consider all features to be [M+H]+, two

adducts of the same molecule might be considered as different moieties, given that fragmentation patterns will differ, even if no difference exists. On the same vein, since similarity thresholds of the MS2Mol algorithm are essential for the estimation of diversity, the authors should clearly state how are they calculated in text, not by reference, along with potential limitations.
Finally, I believe the work would greatly benefit from including data on phylogenetics of the samples, adding diversity estimates to their sample and extrapolation data. If, for example, most of the 400,000 plant species are phylogenetically distant from the sampled species, then the reader can reasonably assume that this might be an underestimation of chemical diversity when presented with the evidence. If, on the other hand, the original sample has more diversity than the total number of plant species, this might not be the case. In any case, all of the relevant assumptions should be clearly stated.
Minor note: One of the main arguments for extrapolating the diversity estimate into the rest of the plants comes from Figure 3D, where increasing MS1 adducts increases with number of samples; it would greatly help explaining the difference seen between species if the authors clarify the tissues sampled per species. E.g. if the species that only doubles the number of features contains only aerial and vegetative tissue, compared to the species that increases 6fold which might include root or reproductive tissue, etc. This might also help the authors in justifying the extrapolation of the estimate.

Methods

Are the methods appropriate to the aims of the study, are they well described, and are necessary controls included? Choose an item.

Conclusions

Are the conclusions adequately supported by the data shown? Choose an item.

Reporting Standards

Does the manuscript adhere to the journal's guidelines on minimum standards of reporting? Choose an item.

Choose an item.

Statistics

Are you able to assess all statistics in the manuscript, including the appropriateness of statistical tests used? Choose an item.

Quality of Written English

Please indicate the quality of language in the manuscript: Choose an item.

Declaration of Competing Interests

Please complete a declaration of competing interests, considering the following questions:

- Have you in the past five years received reimbursements, fees, funding, or salary from an organisation that may in any way gain or lose financially from the publication of this manuscript, either now or in the future?
- Do you hold any stocks or shares in an organisation that may in any way gain or lose financially from the publication of this manuscript, either now or in the future?
- Do you hold or are you currently applying for any patents relating to the content of the manuscript?
- Have you received reimbursements, fees, funding, or salary from an organization that holds or has applied for patents relating to the content of the manuscript?
- Do you have any other financial competing interests?
- Do you have any non-financial competing interests in relation to this paper?

If you can answer no to all of the above, write 'I declare that I have no competing interests' below. If your reply is yes to any, please give details below.

I declare that I have no competing interests.

I agree to the open peer review policy of the journal. I understand that my name will be included on my report to the authors and, if the manuscript is accepted for publication, my named report including any attachments I upload will be posted on the website along with the authors' responses. I agree for my report to be made available under an Open Access Creative Commons CC-BY license (http://creativecommons.org/licenses/by/4.0/). I understand that any comments which I do not wish to be included in my named report can be included as confidential comments to the editors, which will not be published.

Choose an item.

To further support our reviewers, we have joined with Publons, where you can gain additional credit to further highlight your hard work (see: https://publons.com/journal/530/gigascience). On publication of this paper, your review will be automatically added to Publons, you can then choose whether or not to claim your Publons credit. I understand this statement.

Yes Choose an item.
