# Supplementary material for: Defining the limits of plant chemical space: challenges and estimations
Source: Gigascience. 2025 Apr 4;14:giaf033. doi: 10.1093/gigascience/giaf033 (PMC11970369; doi:10.1093/gigascience/giaf033)
Supplement: giaf033_Reviewer_2_Report_Original_Submission — Kohulan Rajan -- 2/24/2025 [file giaf033_reviewer_2_report_original_submission.pdf]

Reviewer Report

Title: Defining the limits of plant chemical space: challenges and estimations

Version: Original Submission **Date:** 2/24/2025

Reviewer name: Kohulan Rajan

Reviewer Comments to Author:

Review: Defining the limits of plant chemical space: challenges and estimations
This work presents an important contribution to understanding the chemical diversity of plants through a systematic analysis combining metabolomics data and literature mining. The authors address a question in the field and employs multiple complementary approaches to estimate the size of the plant chemical space.
Here are my few suggestions and question to the authors to clarify,
1. When introducing an abbreviation one could use caption letters "Natural Products (NP)"
2. There is no list of abbreviations in the document, so introduce them first and then use them. There may be some readers who are unfamiliar with the terms COCONUT and LOTUS.
3. Is there any prior work using similar combined metabolomics/literature approaches to estimate plant chemical space? If so, these should be cited. If not, please state this explicitly to highlight the novelty of your method.
4. Cite SMILES
5. While the paper describes the use of 'literature datasets,' it appears that only existing databases (COCONUT and LOTUS) are being utilized. It would be helpful if authors could clarify whether any direct literature mining was conducted. If not, consider revising terminology to more accurately reflect the use of curated databases rather than primary literature sources.
6. Great to see the data and code openly shared on both Zenodo and GitHub. I also find the GitHub repository very useful with regard to all the provided notebooks. To maximize reusability, please consider adding a detailed "How to Use" section to the README that guides others in replicating or building upon this work.
7. The different clustering thresholds (0.7 vs 0.8) lead to notably different estimates. Could you discuss which threshold might be more appropriate for this specific application to plant metabolomics data?

Methods

Are the methods appropriate to the aims of the study, are they well described, and are necessary controls included? Choose an item.

Conclusions

Are the conclusions adequately supported by the data shown? Choose an item.

Reporting Standards

Does the manuscript adhere to the journal's guidelines on [minimum standards of reporting?] Choose an item.

Choose an item.

Statistics

Are you able to assess all statistics in the manuscript, including the appropriateness of statistical tests used? Choose an item.

Quality of Written English

Please indicate the quality of language in the manuscript: Choose an item.

Declaration of Competing Interests

Please complete a declaration of competing interests, considering the following questions:

- Have you in the past five years received reimbursements, fees, funding, or salary from an organisation that may in any way gain or lose financially from the publication of this manuscript, either now or in the future?
- Do you hold any stocks or shares in an organisation that may in any way gain or lose financially from the publication of this manuscript, either now or in the future?
- Do you hold or are you currently applying for any patents relating to the content of the manuscript?
- Have you received reimbursements, fees, funding, or salary from an organization that holds or has applied for patents relating to the content of the manuscript?
- Do you have any other financial competing interests?
- Do you have any non-financial competing interests in relation to this paper?

If you can answer no to all of the above, write 'I declare that I have no competing interests' below. If your reply is yes to any, please give details below.

declare that I have no competing interests

I agree to the open peer review policy of the journal. I understand that my name will be included on my report to the authors and, if the manuscript is accepted for publication, my named report including any attachments I upload will be posted on the website along with the authors' responses. I agree for my report to be made available under an Open Access Creative Commons CC-BY license (http://creativecommons.org/licenses/by/4.0/). I understand that any comments which I do not wish to be included in my named report can be included as confidential comments to the editors, which will not be published.

Choose an item.

To further support our reviewers, we have joined with Publons, where you can gain additional credit to further highlight your hard work (see: https://publons.com/journal/530/gigascience). On publication of this paper, your review will be automatically added to Publons, you can then choose whether or not to claim your Publons credit. I understand this statement.

Yes Choose an item.
